# Central New York Homœopathic Medical Society

**Published:** 1887-03

**Authors:** Julius Schmitt

**Affiliations:** The Secretary


### CENTRAL NEW YORK HOMŒOPATHIC MEDICAL SOCIETY.

REPORT OF PROCEEDINGS BY THE SECRETARY, JULIUS SCHMITT, M. D.

ROCHESTER, N. Y., December 16th, 1886,
DR. ALLEN B. CARR'S OFFICE.

Dr. J. A. Biegler called the meeting to order at half-past ten o'clock A. M.

The following members answered to the roll-call: J. A. Biegler, E. P. Hussey, Stowe, Baker, Clapp, Carr, Voak, Hermance, Schmitt.

The proceedings of the last meeting were read and adopted.

Dr. Stowe moved as the next order of business the report of the Committee on By-Laws. Carried.

Dr. J. A. Biegler presented, as the work of the Committee, the following By-Laws, which were read and discussed and adopted as follows:

### BY-LAWS.

### SECTION I.

This Society shall hold at least four meetings in each year. The annual meeting shall be held at the city of Syracuse, on the third Thursday in September, and the other meetings shall be quarterly, and held at the locality named by the Society at a previous meeting, providing it be within the territory of counties heretofore named in the Constitution.

### SECTION II.

The officers of this Society shall remain in office until others are chosen.

### SECTION III.

The President shall preside at all meetings of the Society, preserve order, put all questions, announce the decisions, and appoint all committees not otherwise ordered. In his absence the Vice-President shall perform the same duties.

### SECTION IV.

The Secretary shall perform all the duties incident to such office; also all the duties pertaining to the office of Treasurer.

### Section V.

Applications for membership may be received at any regular meeting, and they must be indorsed by three members of this Society who are in good standing; said indorsement shall not be made alone upon the general reputation of applicants, but at least one of the members, who make said indorsement, must attest, *of his own knowledge*, the integrity of the practice of said applicant.

### Section VI.

Applications received in accordance with Section V shall be in the keeping of the Chairman of the Board of Censors for the period of six months, and it shall be the duty of said Chairman to furnish a list of the names of applicants to each member of the Society who shall be in good standing as soon as practicable after the meeting at which they were presented. The members who receive such lists shall return them to the Chairman of the Board of Censors, with their vote—yes or no—after the name of each applicant they may see fit to vote upon, not later than one month prior to the meeting at which they shall be submitted to ballot. A majority of the votes so received shall enable the Board of Censors, through their Chairman, to report favorably or otherwise.

### Section VII.

At the next meeting after the expiration of six months from the time applications have been received, and upon the recommendation of the Chairman of the Board of Censors, in accordance with Section IV, an election by ballot shall be held to decide the eligibility of each applicant, and a two-thirds majority of the members present shall be necessary to elect. If an applicant be not elected, he may, upon a majority vote, if he so desire, receive a second ballot at a regular meeting not under the period of one year.

### Section VIII.

Every member on his or her election shall pay to the Treasurer the sum of one dollar, and one dollar annually thereafter.

### Section IX.

Any member who shall fail to pay his annual dues, shall, for the time during which they shall remain unpaid, forfeit all privileges of membership, except by unanimous consent of members present.

SECTION X.

The By-Laws may be amended at any regular meeting of the Association by a two-thirds vote of the members present; notice of the proposed change having been served upon every member at least three months prior to the meeting at which it is brought up for a vote.

SECTION XI.

At each regular meeting a subject for discussion shall be named by the Association for the next meeting, and it shall be the duty of the President to require one or more sections of the *Organon* to be read. Also, each member shall be required to read an article or report a case in writing at least once a year.

SECTION XII.

At all meetings of the Society the following shall be the order of business:

1. Calling of the roll.
2. Reading of the minutes of the last meeting.
3. Communications from the President.
4. Report of Censors and election of new members.
5. Reading of the *Organon*.
6. Reports on medical subjects.
7. Miscellaneous business.
8. Report of the Secretary and Treasurer.
9. Election of officers (at the annual meeting).
10. Adjournment.

Dr. Hussey moved that the President appoint a Board of three Censors, to stand until the next annual meeting. Carried.

Dr. Biegler appointed as such Board, Drs. Hussey, Hawley, and Nash, Dr. Hussey to act as chairman.

Dr. Carr moved that the President and Secretary be made a Committee to publish the Constitution and By-Laws, and that the Committee have power to act. Carried.

Dr. Stowe also moved that the declaration of principles be printed with the new Constitution. Carried.

Dr. Stowe moved that the President appoint a Committee of three to draw up a memorial of the late Dr. R. R. Gregg, of Buffalo, a deceased member of this Society. Carried.

The President appointed as such Committee Drs. Stowe, Hussey, and Hawley.

At one and a half P. M. the Society adjourned to accept the hospitality of Dr. Carr.

The meeting was again called to order by the President at two and a half P. M., when the subject for discussion, Sycosis, was taken up. Dr. Schmitt read the following paper on Sycosis.

### "SYCOSIS."

JULIUS SCHMITT, M. D., ROCHESTER, N. Y.

